# Simultaneous mitigation of 4(5)‐methylimidazole, acrylamide, and 5‐hydroxymethylfurfural in ammonia biscuits by supplementing with food hydrocolloids

**DOI:** 10.1002/fsn3.1250

**Published:** 2019-11-07

**Authors:** Rasha M. A. Mousa

**Affiliations:** ^1^ Home Economic Department Faculty of Specific Education Assiut University Assiut Egypt; ^2^ Biochemistry Department Faculty of Science University of Jeddah Jeddah Saudi Arabia

**Keywords:** 4(5)‐methylimidazole, 5‐hydroxymethylfurfural, acrylamide, ammonia biscuits, Hydrocolloids

## Abstract

In the current work, methodological approach for the incorporation of food hydrocolloids gum Arabic (GA), pectin, and carboxymethylcellulose (CMC) in biscuit dough was firstly investigated to mitigate simultaneously the formation of 4(5)‐methylimidazole (4(5)‐MI), acrylamide (AA), and 5‐hydroxymethylfurfural (5‐HMF) in ammonia biscuits. Results revealed that the percent inhibition of 4(5)‐MI was ranged from 50.5% to 89.9% by increasing the GA amount from 0.01 g to 0.05 g, respectively. Furthermore, the use of 0.05 g GA reduced significantly AA content up to 58.6% compared to the control biscuits. Moreover, the highest inhibition of 5‐HMF with 74% depression was achieved by 0.05 g GA. Reasons could be referred to the formation of GA layer on the surface of biscuits. This was confirmed by scanning electron microscope and water loss analysis. Additionally, browning intensity and sensory analysis showed that the supplementation with GA had preserved the quality and consumers’ overall acceptability of ammonia biscuits.

## Graphical abstract

In the current work, methodological approach for the incorporation of food hydrocolloids gum Arabic (GA), pectin, and carboxymethylcellulose (CMC) in biscuit dough was firstly investigated to mitigate simultaneously the formation of 4(5)‐methylimidazole (4(5)‐MI), acrylamide (AA), and 5‐hydroxymethylfurfural (5‐HMF) in ammonia biscuits. This was confirmed by scanning electron microscope and water loss analysis. Additionally, browning intensity, mineral and sensory analysis showed that the supplementation with GA had preserved the quality and consumers' overall acceptability of ammonia biscuits.

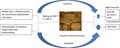

## 1 INTRODUCTION

Ammonia biscuits are one of the most marketable bakery products and internationally get ready as described in the American Association of Cereal Chemists (AACC, 1995) from a dough whose main ingredients are from wheat flour, refined sucrose, oil, ammonium bicarbonate, and water. Then, all the above ingredients were thoroughly mixed and were baked at high temperatures. This preparation process was useful for producing acceptable flavor, color, and aroma properties (Mousa, 2014; Youssef & Mousa, 2012). However, recently one of the important consequences of this preparation process is the production of particular compounds, particularly during baking and raising health harm, such as 4(5)‐methylimidazole (4(5)‐MI), acrylamide (AA), and 5‐hydroxymethylfurfural (5‐HMF) (Jung, Kim, & Lee, 2017; Mogol & Gökmen, 2016; Nguyen, Fels‐Klerx, & van Boekel, 2017; Stadler et al., 2002; Wu, Kong, Huang, & Yu, 2015; Wu, Yu, Kong, & Yu, 2016). Some modifications were previously suggested in the preparation of ammonia biscuits in order to improve their nutritional value; however, in all trials the formation of 4(5)‐MI, AA, and 5‐HMF is still a challenge.

4(5)‐MI (nitrogen heterocyclic compound) was recently reckoned by researchers as possibly carcinogenic group 2B compound by the International Agency for Research on Cancer (IARC) Monographs (IARC, 2018). In addition, the toxicity of 4(5)‐MI was reported by the European Food Safety Authority (EFSA) (EFSA, 2011) due to its increased risk of lung tumors in mice. 4(5)‐MI was reported to be formed in the class III caramel process by heating glucose/fructose and ammonia containing compounds at high temperatures (Wu et al., 2015, 2016). In addition, AA is a prospect carcinogen and genotoxin compound resulting from heating asparagine with glucose/fructose (Maillard reaction) (Mousa, 2018). Furthermore, 5‐HMF (oxygen heterocyclic compound) was declared as cytotoxic compound bringing about irritation to skin, eyes, and upper respiratory tract (Morales, 2009). Studies on laboratory animals showed also that 5‐HMF could be potentially carcinogenic compound (Kowalski, Lukasiewicz, Duda‐Chodak, & Zięć, 2013). One of the most common pathways for the formation of 5‐HMF is caramelization process involving the dehydration of aliphatic monosaccharides, glucose and fructose, at high temperatures (Kroh, 1994; Román‐Leshkov, Chheda, & Dumesic, 2006).

In the last few years, several works confirmed the formation of 4(5)‐MI, AA, and 5‐HMF in ammonia biscuits after baking at 180 ᵒC or 200 ᵒC for a period within 10–20 min. During this process, sucrose thermally degrades into free glucose and fructose which subsequently reacts with ammonium bicarbonate producing 4(5)‐MI in levels ranged from 70 to 1,260 μg/kg (Jung et al., 2017). Furthermore, AA forms initially by reacting free asparagine (as a component of wheat flour) with fructose during baking time producing the Schiff base that underwent decarboxylation to form AA in contents ranged from 10 to 200 μg/kg (Mogol & Gökmen, 2016). However, AA content is very sensitive to free asparagine content in wheat flour which mainly depends on its growing and fertilizing conditions. AA could be produced with levels from 600 to 900 μg/kg in wheat cultivated under normal sulfate fertilization; however, more levels of AA between 2,600 and 5,200 μg/kg were also obtained in sulfate‐deprived wheat after heating at 160ᵒC for 20 min (Muttucumaru et al., 2006). On the same hand, in ammonia biscuits, the enolization of glucose is known as the de Bruijn van Eckenstein rearrangement followed by dehydration or β‐elimination of water giving 3‐deoxyhexosulose and further its intramolecular cyclization concurrently with its dehydration produced 5‐HMF (Román‐Leshkov et al., 2006). It was also reported that the decomposition of sucrose during baking of biscuits released a free glucose and fructofuranosyl cation which rapidly dehydrated to form 5‐HMF at 550–3530 μg/kg (Locas & Yaylayan, 2008; Mogol & Gökmen, 2016).

As 4(5)‐MI, AA, and 5‐HMF toxicants formed spontaneously during the baking of ammonia biscuits, some studies were recently conducted to reduce the formation of these compounds by adding compounds such as iron sulfate, magnesium sulfate, zinc sulfate, tryptophan, cysteine, formic acid, and chitosan (Mogol & Gökmen, 2016; Seo, Ka, & Lee, 2014). It was found that iron sulfate awarded the greatest reduction (about 80%) for 4(5)‐MI in biscuits. Otherwise, formic acid decreased significantly the initial rate of AA formation in biscuits to about four times. The 5‐HMF content was slightly decreased in the presence of acids due to its acidic‐catalyzed degradation. However, these additives in ammonia biscuits could significantly affect their sensorial quality.

Food hydrocolloids gum Arabic (GA), pectin, and sodium carboxymethyl cellulose (CMC) are commonly used as negatively charged water‐soluble polymers and used in low amounts as food additives (Mousa, 2018). These food hydrocolloids had distinguished properties coming from high solubility in water, rigid thin film formation or emulsification and biocompatible. Additionally, they could have antioxidant properties attributed to the existence of stable amino acids in their building structures. Therefore, food hydrocolloids have the ability to form tight thin layer with antioxidant merit during the initial stages of baking for ammonia biscuits. Subsequently, its stable surface structure on biscuits could significantly inhibit or interfere with the factors accelerating the formation of toxicants. However, there is scarce information about the effect of use of food hydrocolloids on the quality of ammonia biscuits.

Therefore, the current work aimed to investigate the influence of incorporation of three types of food hydrocolloids GA, pectin, and CMC with variable concentrations on the simultaneous mitigation of carcinogenic compounds 4(5)‐MI, AA, and 5‐HMF in ammonia biscuits. Furthermore, the quality and sensorial acceptability of new biscuit formulations was also investigated. The obtained results would be of great benefit to home workers, researchers, and food industry.

## 2 MATERIALS AND METHODS

### 2.1 Chemicals and solutions

AA (≥99.8% in purity), 4(5)‐MI (*>*98.0% in purity), and 5‐HMF (≥99.0% in purity) were purchased from Merck (Steinheim, Germany). Gum Arabic (GA, *Acacia Senegal*, 8.33% polysaccharide and 2.41% protein), pectin (24% degree of esterification (DE) and MW 112 kDa), sodium carboxymethyl cellulose (CMC, MW 771 kDa), glucose, fructose, asparagine, and bis‐2‐ethylhexylphosphate (BEHPA, *>*97%) were obtained from Sigma‐Aldrich (St. Louis, MO, USA). Isobutyl chloroformate (IBCF, 98%) was purchased from Wako Pure Chemical Industries, Ltd. (Tokyo, Japan). Refined wheat flour was obtained from the local market, and its chemical composition was confirmed with 9.6% protein, 74.3% carbohydrates, 0.8% fat, 0.5% minerals, and 14.9% moisture. Palm oil was purchased from local market, and its chemical composition was confirmed with saponification value 198, iodine value 52, palmitic acid 42.3%, oleic acid 43.0%, stearic acid 5.0%, and linoleic acid 9.9%. In addition, powdered sucrose (Nile Sugar Company, Egypt, pure and melting point 186°C), sodium chloride, sodium bicarbonate, and ammonium bicarbonate were procured from local market. Carrez I and Carrez II solutions were prepared by dissolving about 30.0 g of potassium hexacyanoferrate in 1 L water and about 60.0 g of zinc sulfate in I L water, respectively. Acetic acid, nitric acid, perchloric acid, formic acid, hydrochloric acid, acetonitrile, ethanol, methanol, isooctane, isobutyl alcohol, chloroform, pyridine, potassium phosphate monobasic, and potassium phosphate dibasic were obtained from Fisher Scientific Co. (Tustin, Canada). All solid‐phase cartridges and columns were supplied by Waters Corporation (Ireland). Millipore water system (Millipore, Billerica, MA) was used to prepare the deionized pure water.

### 2.2 Preparation of ammonia biscuits

For the preparation of biscuits dough, 20.0 g of palm oil was creamed with 35.0 g powdered sucrose, 1.0 g NaCl and 0.8 g of NaHCO_3_ for 3 min at 60 rpm using a dough‐mixer Artisan Kitchen Aid 5KSM150 (MI, USA). For each mixing step, the remained mixture was scratched of the bowl sides in order to retain the ingredients as much as possible. Then, 0.4 g of NH_4_HCO_3_ and 17.6 ml of water were added and mixed for 3 min at 150 rpm. After that, 80.0 g of refined wheat flour was added slowly to the mixture and was again mixed for 2 min at 80 rpm to obtain homogenous dough (control biscuit). For hydrocolloids containing biscuits, water in the control biscuits was replaced with variant concentrations of GA, pectin, and CMC dissolved in water (nine treated biscuit samples as shown in Table 1), and subsequently, the other steps were followed as described above. Then, the obtained dough in each sample was rolled in 3 mm thickness using Atlas Brand rolling machine, and subsequently, the sheeted dough was cut into round shape using a 50‐mm‐diameter cutter. Finally, the obtained dough pieces were baked on an aluminum plate in electric oven (Memmert UNE 400, Germany) at two different temperatures 180°C and 200°C for 10 min. Under these conditions, all the proposed biscuit samples had a fully baked crumb without unacceptable too over‐baked conditions. All the obtained biscuits were cooled for 30 min, were packed in low‐density polyethylene bags, and then were stored under desiccation prior to subsequent chemical analysis.

**Table 1:** The studied ammonia biscuit samples blended with variant concentrations of GA, pectin, and CMC food hydrocolloids

Samples	Usage levels (g/ 17.6 ml water)		
	GA	Pectin	CMC
**Control**	0.00	0.00	0.00
**G1**	0.01	‐‐‐	‐‐‐
**G2**	0.05	‐‐‐	‐‐‐
**G3**	0.10	‐‐‐	‐‐‐
**P1**	‐‐‐	0.10	‐‐‐
**P2**	‐‐‐	0.30	‐‐‐
**P3**	‐‐‐	0.50	‐‐‐
**C1**	‐‐‐	‐‐‐	0.10
**C2**	‐‐‐	‐‐‐	0.30
**C3**	‐‐‐	‐‐‐	0.50

### 2.3 Proximate and structural evaluation of ammonia biscuits

Proximate analysis of proposed baked ammonia biscuits was based on the standard method of AOAC (AOAC, 1995) including moisture, crude fat, crude fiber, crude protein, and ash content. Total carbohydrate was calculated from the sum of moisture, crude fat, crude protein, ash, and crude fiber, and lastly subtracting it from 100. The caloric value was calculated using values of 4.0 k.cal/g of protein, 4.0 k.cal/g of carbohydrate, and 9.0 k.cal/g of fat as described elsewhere (Livesy, 1995).

The microstructure of all studied baked biscuits was investigated by scanning electron microscopy (*SEM*) after mounting on individual metallic stubs and sputtering with a Balzers SCD 030 conductive coating of gold (Balzers Union LTD. Liechtenstein). The images of all studies were obtained by a JEOL6060LV variable pressure *SEM* instrument (Jeol (UK) Ltd.). Electron micrographs were produced for cross section of each biscuit type at several different magnifications.

### 2.4 Determination of 4(5)‐MI in ammonia biscuits by GC/MS

The analytical method reported in reference (Jung et al., 2017) was followed to analyze 4‐MI in biscuits with little modifications. Two grams of biscuits was dissolved in a mixture containing 4 ml of phosphate buffer (pH 6.6) and 8 ml of 0.1 mol/L BEHPA in chloroform. Then, the sample solution was centrifuged at 8,000 rpm for 7 min followed by filtration of bottom layer (chloroform phase) through Whatman® No. 2 filter paper. Four milliliters of 0.1 mol/L HCl was added to 6 ml of filtrate followed by shaking for 6 min. Centrifugation of the obtained solution was performed at 3,131 x g for 5 min. Then, 1 ml of aqueous phase (upper layer) was transferred to another vial followed by adding 1 ml of a mixture of acetonitrile, isobutyl alcohol, and pyridine (50:30:20, v/v) and 120 μL of IBCF. After that, 500 μL of 1.0 mol/L sodium bicarbonate solution and isooctane was also added to the mixture and was shaken by hand for 5 s. Finally, 2 μL of the top layer was injected into the GC‐MS instrument in the splitless mode at a temperature of 275°C (Agilent 7820A gas chromatograph and 5977E mass spectrometer, Palo Alto, Calif., USA). The ion source and quadrupole were set at 235 and 160°C, respectively. An electron impact at 70 eV was used for the mass spectrometer in the selective ion monitoring mode. A HP‐5MS column (30 m × 0.25 mm, 0.25 μm) was used as the stationary phase. Helium was selected as a carrier gas at 1.0 ml/min flow rate. The GC oven temperature program was set in steps starting from 80°C (1 min), ramped to 280°C at 30°C/min, and held for 2 min at 280°C. Ions at *m/z* 81, 82, 109, and 182 were selected for 4(5)‐MI detection, and *m/z* 182 was chosen for its quantification. A stock solution of 4(5)‐MI was prepared in 0.1 mol/L HCl to a concentration of 1 mg/ml. Working solutions were prepared by diluting the stock solution to the concentrations between 50 and 3,000 ng/ml. The limit of detection (LOD, 3.3 *SD*/slope) and the limit of quantitation (LOQ, 10 *SD*/slope) of 4(5)‐MI were 19.0 ng/ml and 53.0 ng/ml, respectively. All 4(5)‐MI measurements were repeated three times.

### 2.5 Determination of AA in ammonia biscuits by LC/MS/MS

AA was extracted from ammonia biscuit formulations by following the procedure as described elsewhere (Mogol & Gökmen, 2016, and Mousa, 2018). An amount of each biscuit formulation (1.0 gram) was dissolved in a solution mixture containing 9.0 ml of 10 mmol/L formic acid, 0.5 ml of Carrez I, and 0.5 ml of Carrez II. The supernatant of each sample solution was collected by centrifugation at 3,131 × g for 7 min. Then, the extractions were repeated two times using 5 ml of 10 mmol/L formic acid as extraction solvent followed by centrifugation to achieve a clear extract. Further cleaning up of the extract was carried out by passing 1.5 ml of the supernatant through a preconditioned Oasis MCX cartridge. Then, the eluent was filtered through 0.45‐μm membrane filter and injected into LC/MS/MS. An Agilent 1,100 HPLC system equipped with a binary pump and coupled to an Agilent 1,100 MS detector (Waldbronn, Germany) was used to carry out the LC/MS/MS analysis. The mode atmospheric pressure chemical ionization (APCI) was chosen for the AA analysis. The prepacked SunFire^TM^ C18 column (250 × 4.6 mm, 5 µm, Waters Corporation, Ireland) was used as the stationary phase, and an isocratic mixture of 0.01 mmol/L acetic acid in a 0.2% formic acid was used as the mobile phase at 0.8 ml/min flow rate. Data acquisition was carried out in the selected ion monitoring (SIM) mode using the following parameters: nitrogen drying gas (100 psi), nebulizer pressure of 60 psi, vaporizer temperature at 425°C, drying gas temperatures at 325°C, capillary voltage of 4 kV, corona current of 4 µA, and fragmented voltage of 55 eV. The ions monitored for AA were *m/z* 55 and 72, and the* m/z* 72 was selected for its quantification. Working solutions of AA were prepared by diluting its stock solution (1 mg/ml) in water to the range between 1.0 and 200.0 ng/ml. LOD and LOQ of AA were 0.2 ng/ml and 0.8 ng/ml, respectively. All AA analyses were conducted in triplicate.

### 2.6 Determination of 5‐HMF in ammonia biscuits by HPLC

5‐HMF was extracted from ammonia biscuit formulations by following the procedure as described elsewhere (Mogol & Gökmen, 2016). An amount of each biscuit formulation (1.0 gram) was dissolved in a solution mixture containing 9.0 ml of 10 mmol/L formic acid, 0.5 ml of Carrez I, and 0.5 ml of Carrez II. The supernatant of each sample solution was collected by centrifugation at 8,000 rpm for 7 min. Then, the extractions were repeated two times using 5 ml of 10 mmol/L formic acid as extraction solvent followed by centrifugation to achieve a clear extract. Further cleaning up of the extract was carried out by passing 1.5 ml of the supernatant through a preconditioned Oasis MCX cartridge. The extract was then injected onto an Agilent 1,100 HPLC system (Waldbronn, Germany) consisting of a quaternary pump and a diode array detector. The chromatographic separations were carried out on an Inertsil ODS‐3 column (4.6 × 250 mm, 5 μm) as a stationary phase, and the mobile phase was an isocratic mixture of 10 mmol/L formic acid and acetonitrile (85:15, v/v) at 1.0 ml/min flow rate, 25°C an oven temperature, and 285 nm detection wavelength. Working standards of 5‐HMF were daily prepared by diluting the stock solution (1.0 mg/ml) in water to the range between 0.1 and 50 ng/ml. The LOD and LOQ of 5‐HMF were 0.01 ng/ml and 0.09 ng/ml, respectively. All 5‐HMF analyses were conducted in triplicate.

### 2.7 Water loss (WL) and browning intensity in ammonia biscuits

Water contents (WCs) of all studied biscuit samples were measured on the dried basis before and after baking process (AOAC, 2005). In these experiments, samples were put in metal plates and then were dried in a forced air oven at 180°C. The following equation is used for the calculation of water loss percent as the following:

$$\begin{array}{cc} & \%\text{Water loss}\left(\text{WL}\right)\\ & \;=\left(\left(\text{WC before baking}-\text{WC after baking}\right)/\text{WC before baking}\right)x100 \end{array}$$

All results were expressed as mean values of the original samples percent (w/w, %) after three replications.

A baking contrast meter (Konica Minolta Colorimeter BC‐10) was used for the determination of browning intensity in the studied ammonia biscuits (Nguyen et al., 2017). The contrast meter ranged from 0 for the darkest to 5.25 for the lightest color. Color measurement was repeated three times in the middle of each biscuit.

### 2.8 Determination of glucose, fructose, and asparagine in wheat flour

In order to determine the amount of glucose and fructose in wheat flour, the procedure described in Nguyen et al. (2017) was followed with small modifications. Two grams of wheat flour was dissolved in 25 ml of water–ethanol mixture (1:1, v/v). The mixture was shaken for 2 min, incubated for 1.5 hr at 40°C, and shaken again for 2 min. The mixture was then cooled at −20°C for 30 min and centrifuged at 4°C for 8 min with 8,000 rpm. Next, the separated supernatant was centrifuged again under the same conditions followed by filtration through a 0.2‐µm filter and finally injected into HPLC system equipped with an evaporative light scattering (ELS) detector. A stationary phase of Alltech Prevail Carbohydrate ES column (250 × 4.6 mm, 5 µm) was used for the separation of analytes. The mobile phase was acetonitrile–water (75:25 v/v) at fixed flow rate of 1 ml/min and fixed wavelength of 220 nm. Stock solutions of glucose and fructose were prepared at a level of 1.0 mg/ml in water. Working standards were daily prepared in water to give the following concentration ranges 1–1000 and 1.5–1000 ng/ml for glucose and fructose, respectively. The LOD and LOQ of glucose were 0.01 ng/ml and 0.09 ng/ml, respectively. In addition, the LOD and LOQ values of fructose were 0.04 ng/ml and 1.5 ng/ml, respectively. The analysis of glucose and fructose in wheat flour was performed once.

In order to determine asparagine, a sample solution was prepared by weighing 1 g of flour in 25 ml of water. The sample solution was shaken for 2 min and incubated for 1 hr at 25°C followed by cooling at −20°C for 20 min and centrifuged at 4°C for 8 min with 8,000 rpm. Next, the separated supernatant was centrifuged again for 10 min followed by passing through a 0.45‐µm filter and finally injected into LC/MS/MS system equipped with a negative ion mode. A stationary phase of Hypercarb column (100 × 3 mm, 5 µm) was used. The mobile phase (A) was 100% water, and mobile phase (B) was 100% methanol. The gradient run started with 100% water for 6 min, and 100% methanol was set for 1 min. After that, the mobile phase was set back to 100% water. The flow rate was fixed at 0.4 ml/min. Working standards of asparagine were daily prepared by diluting the stock solution (1.0 mg/ml) in water to the concentration range between 1.0 and 500.0 ng/ml. The LOD and LOQ values of asparagine were 0.2 ng/ml and 1.0 ng/ml, respectively. The analysis of asparagine in wheat flour was performed once.

### 2.9 Hedonic evaluation test

The proposed different types of ammonia biscuits were subjected to consumers’ acceptability using hedonic test (Birol, Meenakshi, Oparinde, Perez, & Tomlins, 2015). The panelist is composed of students and collaborators that are usually consuming ammonia biscuits (*n* = 50). They are free from sinus problem or cold flue during evaluation period. Ten panelists involved daily for 4‐day evaluation of 15 different samples distributed in random arrangements among evaluators at a time under “daylight” illumination and in isolated bowls. Control samples were initially presented for each evaluator. Mean value of each sample was recorded after three evaluations. Samples were served in white background paper plates which identified by random three digits. Panelists were then asked to write their degree of liking on a paper ballot with a 9‐point hedonic rating scale (Mousa, 2018). As usual, color, appearance, aroma, texture, taste, and overall acceptability were evaluated within this test. Evaluators instructed to clean their palate with water among sample evaluations.

### 2.10 Statistical Analysis

The data analysis for each set was carried out after repeating measurements of each test three times in the presence of one control. Mean values were expressed as means ± standard deviation. Minitab statistical software (USA) was used to apply the analysis of variance. A significance level of *p* < .05 using the Tukey's range test was used for presenting the significance of differences among values.

## 3 RESULTS AND DISCUSSION

Ammonia biscuit samples were currently used as real food complex matrices to test the effect of food hydrocolloids GA, pectin, and CMC at different low amounts on the spontaneous formation of 4(5)‐MI, AA, and 5‐HMF coming from Maillard and caramelization reactions during the baking process.

### 3.1 Reduction of 4(5)‐MI in the studied ammonia biscuits

**Figure 1:**
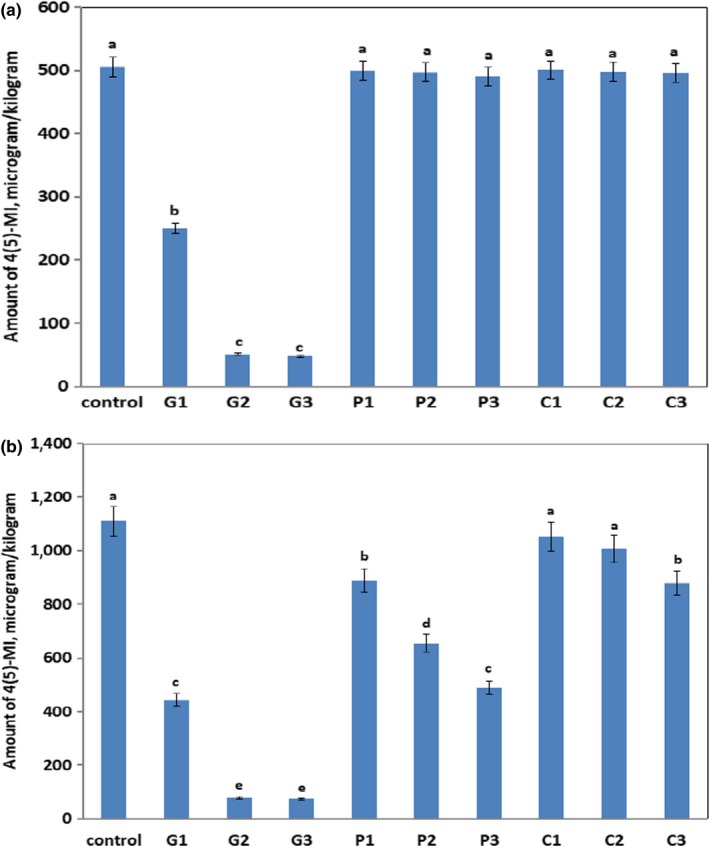
4(5)‐MI content (µg/kg dry weight) of ammonia biscuits baked at 180°C (a) or 200°C (b) for 10 min. Different letters indicate where significant differences occurred (*p* < .05) between the treated samples and control sample at a particular temperature

The formation of 4(5)‐MI in control and treated ammonia biscuits (Table 1) was investigated. In control ammonia biscuits, the amount of 4(5)‐MI was found to be 505 and 1109 μg/kg by baking at 180 and 200°C, respectively, as shown in Figure 1 (A and B). The results showed that 4(5)‐MI formation in ammonia biscuits increased about twice with the increase in temperature from 180°C to 200°C. This revealed the effective role of moisture inside biscuits on the formation of 4(5)‐MI which suggested that the low moisture at 200°C could accelerate the reaction rate of caramelization III process producing more amounts of 4(5)‐MI. However, the addition of GA solutions (G1→G3) to the dough reduced significantly the 4(5)‐MI formation (*p* > .05) at all the temperatures studied as shown in Figure 1a,b. It was obvious that the percent inhibition of 4(5)‐MI amount in biscuits was ranged from 50.5% to 89.9% after baking at 180 °C for 10 min compared to the control biscuits by increasing the GA amount from 0.01 g to 0.05 g (G1→G2 samples). However, the percent inhibition of 4(5)‐MI remained without any change by further increasing of GA amount up to 0.1 g. The same phenomenon was observed after baking at 200 °C with the greatest reduction of 4(5)‐MI from 1109 µg/kg dry weight (control) to 77.6 µg/kg dry weight (G2 sample) by using 0.05 g GA giving about 93% inhibition. On the other hand, food hydrocolloid pectin did not give any significant inhibition for 4(5)‐MI in the treated biscuits (P1→P3) after baking at 180°C as depicted in Figure 1a. However, it is interesting to observe a positive effect of pectin at 200°C baking temperature on the inhibition of 4(5)‐MI in ammonia biscuits. The addition of 0.1 g pectin to the biscuit dough reduced 4(5)‐MI with 20% inhibition (P1 sample) compared to control biscuits, and further inhibition into 55.9% was obtained by adding 0.5 g pectin (P3 sample) as shown in Figure 1b. On the contrary, the addition of CMC solution to the biscuit dough did not significantly affect the 4(5)‐MI formation (*p* > .05) at all temperatures except the addition of 0.5 g CMC/17.6 mL water (C3 sample) reduced slightly the 4(5)‐MI formation after biscuit baking at 200 C with percent inhibition 20.8%. These observations could be attributed to that GA, pectin, and CMC could be used to modify the texture of biscuits by functioning as gelling or thickening agents during baking at high temperatures. This certainly would hold the water capacity inside biscuits and subsequently would affect the course of class III caramelization leading to inhibit the formation of carcinogenic 4(5)‐MI. Therefore, the addition of 0.05 g/ 17.6 mL GA solution to the biscuit dough could significantly (p < 0.05) inhibit 4(5)‐MI in the range from 89.9% to 93% after biscuit baking at 180 °C and 200 °C for 10 min.

### 3.2 Reduction of AA in the studied ammonia biscuits

**Figure 2:**
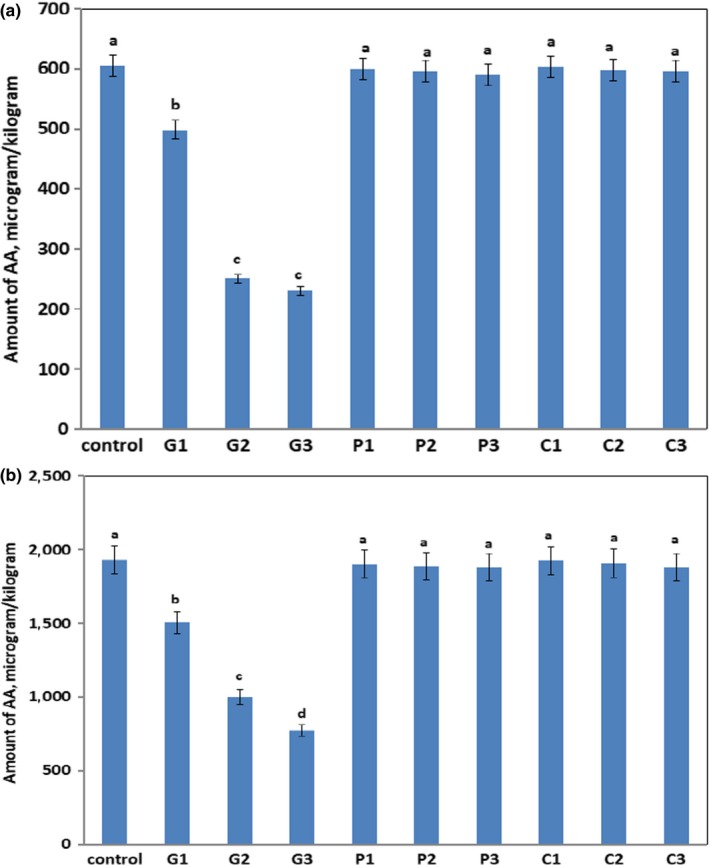
AA content (µg/kg dw) of ammonia biscuits baked at 180°C (a) or 200°C (b) for 10 min. Different letters indicate where significant differences occurred (*p* < .05) between the treated samples and control sample at a particular temperature

The AA content is very sensitive to the amounts of free asparagine (limiting factor) in wheat flour*.* The amount of free asparagine was measured in the wheat flour, and it was found to be 3020.6 mg asparagine/kg wheat flour. Furthermore, fructose and glucose were quantified in wheat flour and were found to be 1020.9 and 420.4 mg/kg, respectively. In the previous study (Surdyk, Rosén, Andersson, & Åman, 2004), it is well known that added fructose increased AA content more than added glucose in heat‐treated food products. Prior to the baking of ammonia biscuits, no AA was detected in the biscuit dough. These results also corroborate with the previous study (Nguyen, Fels‐Klerx, Peters, & Boekel, 2016) in which the compound was detected after 10 min of baking in the ammonia biscuits. In the current work, results in Figure 2a showed that the amount of AA in control biscuit after baking at 180°C for 10 min was 604.3 μg/kg dw. Rising of baking temperature up to 200 °C increased AA content up to 1931.4 μg/kg dry biscuits as shown in Figure 2b, which reflected the direct significant of baking temperature on rising the formation of AA. It is obvious that AA amounts formed in ammonia biscuits at all baking temperatures were higher than the recommended EU benchmark level 500 μg/kg in food (EU, 2017). Figure 2a,b shows that the addition of GA to the biscuit dough (G1→G3) caused a significant inhibition in the formation of AA at all studied temperatures. At 180°C baking temperature, the use of 0.05 g GA/ 17.6 ml water (G2 sample) decreased AA content down to 250.4 μg/kg dry biscuits with 58.6% inhibition compared to the control sample and remained without any further significant change in the presence of 0.1 g GA/ 17.6 ml water (G3 sample). In this case, 0.05 g GA (G2 sample) is significant to reduce AA content lower than the EU‐recommended level in food (EU, 2017). The same observation was happened at 200℃ baking temperature by adding GA to the biscuit dough, but in all cases the AA amount is still higher than the recommended EU level in food (EU, 2017) as indicated in Figure 2b. The reasons for this behavior could be due to the gelling or thickening effect of GA on the texture modification of proposed biscuits which consequently could interfere with the molecular interactions between fructose and asparagine as precursors of AA formation. Moreover, the acidic pH value of GA solution (pH = 4.9) could be another factor to facilitate the reduction of AA formation in biscuits. However, the magnitude of GA influence on the mitigation of AA is lower than 4(5)‐MI as described above. The reasons might be due to the molecular movements and interactions between the GA and AA precursors suffered from some limitations in the chemical composition of wheat flour. The use of pectin and CMC at all concentration levels did not significantly affect the AA formation (*p* > .05) at all temperatures compared to the control biscuits as depicted in Figure 2a,b. Therefore, the addition of 0.05 g GA/ 17.6 mL water to the biscuit dough could significantly (*p* < 0.05) inhibit AA content lower than the EU‐recommended level in food (EU, 2017) after biscuit baking at 180°C for 10 min.

### 3.3 Reduction of 5‐HMF in the studied ammonia biscuits

**Figure 3:**
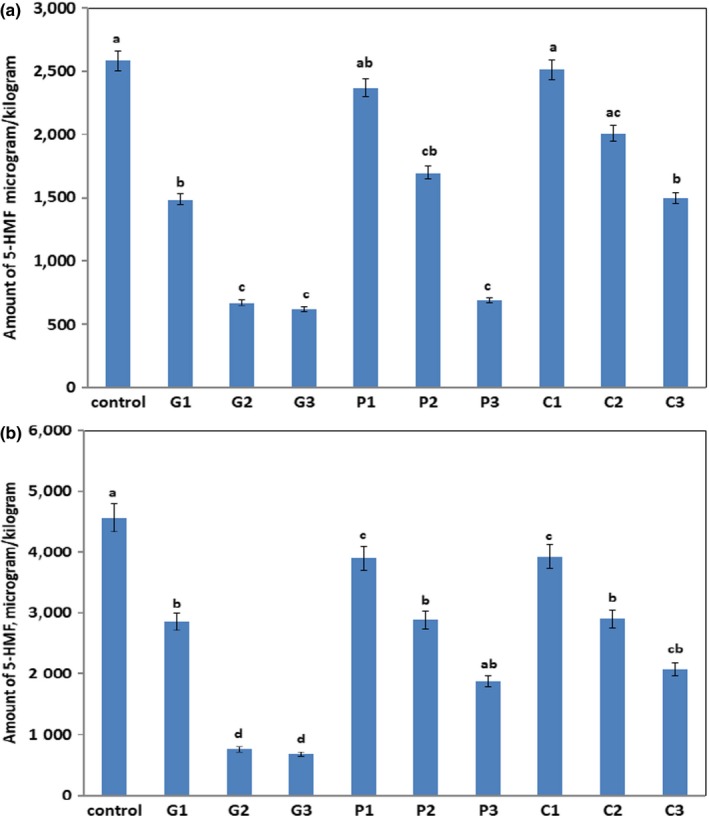
5‐HMF content (µg/kg dw) of ammonia biscuits baked at 180°C (a) or 200°C (b) for 10 min. Different letters indicate where significant differences occurred (*p* < .05) between the treated samples and control sample at a particular temperature

Figure 3a,b shows the 5‐HMF formation in the studied biscuits baked at 180°C and 200°C for 10 min. 5‐HMF formation in control biscuits was found to be 2,581.5 μg/kg dw and 4,567.1 μg/kg dw at 180°C and 200°C, respectively. The addition of GA to the biscuit dough significantly decreased the 5‐HMF formation at all the temperatures studied (*p* < .05). The highest inhibition of 5‐HMF was achieved by using 0.05 g GA with 74% and 83.4% at 180°C and 200°C, respectively. However, the addition of further amounts of GA did not significantly affect the AA formation (*p* > .05) at all temperatures compared to G2 sample. In addition, there was a significant inhibition of 5‐HMF by the addition of pectin and CMC (*p* < .05) at all temperatures compared to the control biscuit sample as shown in Figure 3a,b. However, the inhibition of 5‐HMF was fourfold by GA sample (G2‐sample) compared to the pectin (P2 sample) and CMC (C2 sample). Therefore, the addition of 0.05 g GA water to the biscuit dough could significantly (*p* < .05) inhibit 5‐HMF content at 180°C for 10 min. The previous study stated that the addition of dietary fibers could cause a significant inhibition in the 5‐HMF content of cookies (Mogol & Gökmen, 2016) due to the enhancement of water‐holding capacity of the dough. These results are in a good agreement with the current results. In addition, in the current work, it was observed that the results of 5‐HMF were to some extent close to the results of 4(5)‐MI but showed different trends to the results of AA among the treated biscuits. The main reason might be the effect of moisture evaporation and greatly affected the course of reactions.

### 3.4 Water loss and browning intensity measurement in the studied ammonia biscuits

Changes in water loss (WL) and browning intensity were measured in the nine treated biscuits and compared with the control biscuits. The data revealed that the presence of GA resulted in significant changes (*p* < .05) in comparison with pectin and CMC after baking at 180°C. Water loss (WL) values of GA (G1 → G3 samples) were in the range from 30.3 ± 0.4% to 37.8 ± 0.3% compared to 49.1 ± 0.2% in the control biscuit sample. However, after baking at 200°C, WL values of GA were slightly lower than those values at 180°C to be in the range from 28.1 ± 0.5% to 34.5 ± 0.4%. On the other hand, the WL values using pectin and CMC hydrocolloids did not show any significant difference (*p* > .05) compared to the control biscuit sample after baking at 180°C. However, pectin and CMC gave significant WL values compared to the control sample after biscuit baking at 200°C in the range from 42.3 ± 0.6% to 49.0 ± 0.1%. Similar results for the browning intensity were observed among the studied biscuits. Ammonia biscuits supplemented with GA had the highest browning intensity value, that is, the lightest color. When GA hydrocolloid layer formed after baking at 180°C and 200°C for 10 min, WL of ammonia biscuits decreased markedly because GA tight layer could be formed on the external surface of biscuits during baking process. As a result, this would inhibit both of Maillard reaction and caramelization inside ammonia biscuits. Nguyen et al. (2016) confirmed that water evaporated substantially during the first 10 min of baking and to a lesser extent afterward. Therefore, results could be due to the formation of layer rigid (thermal gelation and/or cross‐linking) network during baking. This layer led to tighten the outer surface of biscuits with fewer voids preventing the escape of water from the porous surface, and the surface permeability was then reduced. In order to confirm this possibility, the scanning electron microscope (*SEM*) photographs of control biscuits and biscuits supplemented with different food hydrocolloids, particularly GA, were measured. The control sample showed extensive cell separation and ruptured cells due to the large void spaces including middle lamella and cell wall damage resulting in ease escape of interior water. In contrast, G2 sample (ammonia biscuits treated with 0.05 g GA) showed a decrease in its cell size with improving its cellular integrity. In addition, there was no disruption of cell wall or delamination of the middle lamella. This means that the coated surface of treated biscuits had less wide punctures with low capillary pressures leading to less evaporation of water.

### 3.5 Proximate chemical composition and caloric value of ammonia biscuits

The mean values of proximate chemical composition and caloric values of biscuits supplemented with hydrocolloids after baking at 180°C for 10 min are given in Table S1. The data of moisture content, crude protein, and crude fiber content (g/ 100 g) revealed that the addition of GA to the biscuit dough showed a significant difference in measurements compared to the control biscuits. The addition of GA in G2 sample significantly increased moisture, protein, and fiber more than 1.7‐, 1.1‐, and fourfold increments compared to the control biscuits, respectively. However, pectin and CMC increased slightly the moisture content and crude fiber values but did not appear any effect on the others. Moreover, the addition of GA, CMC, or pectin did not influence crude fat and ash content. However, these values were used in the calculation of total carbohydrate and caloric values as depicted in Table S1. The carbohydrate content varies from a high value of 74.1 ± 0.1 g/100 g in a control biscuit to a low value of 68.3 ± 0.2 g/100 g in the G2 sample. Furthermore, the use of GA in ammonia biscuits reduced significantly (*p* < .05) the caloric value down to 412.3 ± 0.2 kcal/100 g compared with control biscuits. Therefore, the formulation of ammonia biscuits with GA reduced the energy by 4.5% compared to the control biscuits. The above results proved that the use of GA‐treated biscuits improved also the nutritional profile of control biscuits.

### 3.6 Consumer acceptability

The results of consumers’ acceptability are shown in Table 2. It was found that the different studied ammonia biscuits presented the acceptable scores for all sensory attributes appreciably higher than the minimum acceptability score, that is, 5.0. The overall acceptance among the most studied samples was not significant (*p* > .05), while the control biscuit sample appeared the highest scores in most of sensory attributes and the overall acceptance. Therefore, the overall sensory attributes of treated ammonia biscuits with hydrocolloids were somewhat worse than the control biscuits; however, the results did not indicate any lack of acceptance. The hydrocolloids, particularly GA, had also retained less carcinogenic compounds 4(5)‐MI, AA, and 5‐HMF inside the baked ammonia biscuits.

**Table 2:** Sensory characteristics of the studied hydrocolloid ammonia biscuits

Sample	Color	Appearance	Aroma	Taste	Overall acceptability
**Control**	8.4 ± 1.2^a^	7.6 ± 0.5^a^	7.7 ± 0.6^ab^	8.0 ± 1.3^ac^	7.9 ± 1.0^cb^
**G1**	8.3 ± 1.0^a^	7.5 ± 1.4^a^	7.3 ± 0.4^a^	7.5 ± 1.7^d^	7.2 ± 1.8^cb^
**G2**	8.7 ± 1.5^ab^	7.6 ± 0.9^a^	7.4 ± 1.3^a^	7.6 ± 1.2^d^	7.5 ± 1.1^cb^
**G3**	8.4 ± 1.0^a^	7.5 ± 1.4^a^	7.1 ± 1.0^a^	7.0 ± 1.3^dc^	6.9 ± 1.4^c^
**P1**	7.6 ± 1.2^c^	6.5 ± 1.3^b^	6.8 ± 1.1^b^	6.7 ± 1.3^b^	6.5 ± 1.5^c^
**P2**	7.3 ± 1.0^bc^	6.6 ± 1.9^b^	6.2 ± 1.9^b^	6.3 ± 1.5^b^	6.2 ± 1.9^c^
**P3**	7.4 ± 1.8^bc^	6.8 ± 1.3^ab^	6.1 ± 1.3^b^	6.1 ± 1.0^b^	6.4 ± 0.8^c^
**C1**	7.5 ± 1.0^c^	6.5 ± 1.1^b^	6.4 ± 1.0^b^	6.2 ± 1.2^b^	6.7 ± 1.1^c^
**C2**	7.7 ± 1.0^c^	6.9 ± 1.1^ab^	6.2 ± 1.2^b^	6.3 ± 1.0^b^	6.3 ± 1.5^c^
**C3**	6.5 ± 1.6^b^	6.7 ± 1.7^ab^	6.1 ± 1.5^b^	6.5 ± 1.5^b^	6.7 ± 1.3^c^

Note

Values are mean ± *SD* of three determinations (*n* = 3). Different letters within a column indicate significantly different values (*p* < .05).

## 4 CONCLUSION

The incorporation of food hydrocolloids in low amounts, particularly GA, into ammonia biscuit formulations where they are processed at 180 °C for 10 min reduced significantly the formation of thermal process toxicants 4(5)‐MI, AA, and 5‐HMF. The percent inhibition of 4(5)‐MI was ranged from 50.5% to 89.9% after baking at 180 °C for 10 min by increasing the GA amount from 0.01 g to 0.05 g added to the biscuit dough. Furthermore, the use of 0.05 g GA decreased AA content with 58.6% inhibition compared to the control biscuits. Moreover, the highest inhibition of 5‐HMF was achieved by 0.05 g GA with 74% depression. The reasons could be due to the formation of tight GA layer as a compact barrier to moisture evaporation during baking process. This was confirmed by SEM and WL analysis. As a result, this would inhibit the molecular interactions of Maillard reaction and caramelization inside ammonia biscuits. However, while the modified GA‐ammonia biscuits were evaluated in sensor attributes lower than the control biscuits, they were still overall acceptable.

## CONFLICT OF INTEREST

The author declares that I do not have any conflict of interest.

## ETHICAL STATEMENT

This study does not involve any human or animal testing.
