# Supplementary material for: Simultaneous mitigation of 4(5)‐methylimidazole, acrylamide, and 5‐hydroxymethylfurfural in ammonia biscuits by supplementing with food hydrocolloids
Source: Food Sci Nutr. 2019 Nov 7;7(12):3912–21. doi: 10.1002/fsn3.1250 (PMC6924299; doi:10.1002/fsn3.1250)
Supplement: Supplementary file 1 [file FSN3-7-3912-s001.docx]

**Supplementary Table 1:**

Proximate chemical composition and caloric value (mean value± SD, n=3) of the studied biscuits after baking at 180 °C for 10 min

| **Sample** | **Moisture content**  **(g/100g)** | **Crude protein**  **(g/100 g)** | **Crude fat**  **(g/ 100g)** | **Ash content**  **(g/100 g)** | **Crude fiber**  **(g/100g)** | **Carbohydrates**  **(g/ 100g)** | **Caloric value (kcal/100g)** |
| --- | --- | --- | --- | --- | --- | --- | --- |
| **Control** | 5.3±0.2^a^ | 7.0±0.2^a^ | 11.9±0.1^a^ | 1.5±0.1^a^ | 0.2±0.2^a^ | 74.1±0.1^a^ | 431.5±0.1^a^ |
| **G1** | 8.1±0.3^b^ | 7.1±0.3^a^ | 11.9±0.2^a^ | 1.6±0.2^a^ | 0.5±0.1^c^ | 70.8±0.2^a^ | 418.7±0.1^c^ |
| **G2** | 9.2±0.1^c^ | 7.4±0.2^b^ | 11.8±0.1^a^ | 1.5±0.1^a^ | 0.8±0.1^b^ | 69.3±0.1^b^ | 413.0±0.1^c^ |
| **G3** | 9.5±0.2^c^ | 7.5±0.4^b^ | 11.9±0.1^a^ | 1.4±0.1^a^ | 0.9±0.2^b^ | 68.8±0.2^b^ | 412.3±0.2^c^ |
| **P1** | 5.8±0.1^a^ | 7.1±0.3^a^ | 11.7±0.4^a^ | 1.5±0.2^a^ | 0.2±0.1^a^ | 73.9±0.3^a^ | 429.3±0.3^b^ |
| **P2** | 6.4±0.1^ab^ | 7.3±0.1^b^ | 11.8±0.3^a^ | 1.5±0.1^a^ | 0.4±0.3^c^ | 72.6±0.2^a^ | 425.8±0.2^b^ |
| **P3** | 6.9±0.2^ab^ | 7.4±0.1^b^ | 11.9±0.2^a^ | 1.6±0.1^a^ | 0.6±0.1^c^ | 71.6±0.2^a^ | 423.1±0.3^b^ |
| **C1** | 5.4±0.2^a^ | 7.0±0.4^a^ | 11.9±0.1^a^ | 1.4±0.2^a^ | 0.3±0.3^a^ | 74.0±0.2^a^ | 431.1±0.2^a^ |
| **C2** | 6.0±0.1^ab^ | 7.1±0.1^a^ | 11.7±0.2^a^ | 1.5±0.2^a^ | 0.4±0.5^c^ | 73.3±0.3^a^ | 426.9±0.3^b^ |
| **C3** | 6.3±0.2^ab^ | 7.3±0.4^b^ | 11.7±0.3^a^ | 1.6±0.3^a^ | 0.5±0.2^c^ | 72.6±0.1^a^ | 424.9±0.1^b^ |

Values which don’t share the same letter in each column are significantly different (p < 0.05)
